# The impact of genetic counselling about breast cancer risk on women's risk perceptions and levels of distress

**DOI:** 10.1038/sj.bjc.6690078

**Published:** 1999-02

**Authors:** A Cull, E D C Anderson, S Campbell, J Mackay, E Smyth, M Steel

**Affiliations:** ICRF Medical Oncology Unit, Western General Hospital, Edinburgh, EH4 2XU, UK; SE Scotland Breast Cancer Genetic Counselling and Screening Clinic, 26 Ardmillan Terrace, Edinburgh, EH11 2JL, UK

**Keywords:** breast cancer genetics, risk counselling, psychological distress, risk perception

## Abstract

Women referred to a familial breast cancer clinic completed questionnaires before and after counselling and at annual follow-up to assess their risk estimate and psychological characteristics. The aims were to determine whether those who attended the clinic overestimated their risk or were highly anxious and whether counselling influenced risk estimates and levels of distress. Women (*n* = 450) at this clinic were more likely to underestimate (39%) than overestimate (14%) their risk. Mean trait anxiety scores were higher than general population data (*t* = 4.9, *n* = 1059, *P* < 0.001) but not significantly different from published data from other screening samples. Overestimators (*z* = 5.69, *P* < 0.0001) and underestimators (*z* = –8.01, *P* < 0.0001) reported significantly different risk estimates (i.e. increased accuracy) after counselling, but significant inaccuracies persisted. Over- (*n* = 12) and underestimators (*n* = 60) were still inaccurate in their risk estimates by a factor of 2 after counselling. Thirty per cent of the sample scored above the cut-off (5/6) for case identification on a screening measure for psychological distress, the General Health Questionnaire (GHQ). GHQ scores were significantly lower after counselling (*t* = 3.6, d.f. = 384, *P* = 0.0004) with no evidence of increasing risk estimate causing increased distress. The risk of distress after counselling was greater for younger women and those who were more distressed at first presentation. The counselling offered was effective in increasing the accuracy of risk perceptions without causing distress to those who initially underestimated their risk. It is worrying that inaccuracies persisted, particularly as the demand for service has since reduced the consultation time offered in this clinic. Further work is needed to evaluate alternative models of service delivery using more sophisticated methods of assessing understanding of risk. © 1999 Cancer Research Campaign


*British Journal of Cancer* (1999) **79**(3/4), 501–508
© 1999 Cancer Research Campaign
Article no. bjoc.1998.0078

### The impact of genetic counselling about breast cancer risk on women's risk perceptions and levels of distress

**A Cull[1], EDC Anderson[2], S Campbell[1], J Mackay[2], E Smyth[2] and M Steel[2]**

[1]ICRF Medical Oncology Unit, Western General Hospital, Edinburgh EH4 2XU; [2]SE Scotland Breast Cancer Genetic Counselling and Screening Clinic, 26 Ardmillan Terrace, Edinburgh EH11 2JL, UK

**Summary** Women referred to a familial breast cancer clinic completed questionnaires before and after counselling and at annual follow-up to assess their risk estimate and psychological characteristics. The aims were to determine whether those who attended the clinic overestimated their risk or were highly anxious and whether counselling influenced risk estimates and levels of distress. Women ($n = 450$) at this clinic were more likely to underestimate (39%) than overestimate (14%) their risk. Mean trait anxiety scores were higher than general population data ($t = 4.9$, $n = 1059$, $P < 0.001$) but not significantly different from published data from other screening samples. Overestimators ($z = 5.69$, $P < 0.0001$) and underestimators ($z = -8.01$, $P < 0.0001$) reported significantly different risk estimates (i.e. increased accuracy) after counselling, but significant inaccuracies persisted. Over- ($n = 12$) and underestimators ($n = 60$) were still inaccurate in their risk estimates by a factor of 2 after counselling. Thirty per cent of the sample scored above the cut-off (5/6) for case identification on a screening measure for psychological distress, the General Health Questionnaire (GHQ). GHQ scores were significantly lower after counselling ($t = 3.6$, d.f. = 384, $P = 0.0004$) with no evidence of increasing risk estimate causing increased distress. The risk of distress after counselling was greater for younger women and those who were more distressed at first presentation. The counselling offered was effective in increasing the accuracy of risk perceptions without causing distress to those who initially underestimated their risk. It is worrying that inaccuracies persisted, particularly as the demand for service has since reduced the consultation time offered in this clinic. Further work is needed to evaluate alternative models of service delivery using more sophisticated methods of assessing understanding of risk.

**Keywords:** breast cancer genetics; risk counselling; psychological distress; risk perception

There is a lack of formal scientific evidence on how best to manage women with a family history of breast cancer, in terms both of communicating about their risk of developing the disease and of advising about the optimal risk management strategy. It is vital that services offered to these women are adequately evaluated to inform future practice.

This paper reports data from an ongoing longitudinal study of the knowledge, attitudes and behavioural and emotional responses of women attending a familial breast cancer clinic in SE Scotland. The clinic was established in 1992, and at that time there were no published psychological data available from the small number of similar clinics in the UK. We were aware of the subsequently published assessments from Manchester of women's perceptions of their risk of developing breast cancer (Evans et al, 1993, 1994). The same method of assessing risk perceptions was therefore adopted in this study. In spite of a subsequent proliferation of cancer risk counselling clinics there has remained a dearth of published reports evaluating the services offered.

The concerns when our clinic opened were that the women seeking referral would be characterized by high anxiety and not necessarily at significantly increased risk. A further concern was that the process of counselling about cancer risk would be anxiety provoking, particularly for those who would be told that their risk was greater than they had previously thought. In the current state of knowledge, the information that can be given about individual risk and the efficacy of available risk management strategies is highly probabilistic. It was recognized that this uncertainty could also generate anxiety. Key issues were therefore to assess women's perceptions of their risk of developing breast cancer and the psychological morbidity associated with cancer risk counselling.

This study was conducted against a background of data accruing from the US to show a substantial proportion of women with a family history of breast cancer with significant levels of psychological distress (Kash et al, 1992) and gross overestimates of their own cancer risk (Lerman et al, 1994*a*; Gagnon et al, 1996) even after risk counselling (Lerman et al, 1995). High levels of perceived susceptibility and associated anxiety have been shown to interfere with adherence to recommended surveillance programmes (Kash et al, 1992; Lerman et al, 1993). The concern has also been expressed that some women will deal with their concerns by making ill-considered requests for genetic testing or prophylactic surgery (Lerman et al, 1994*b*). In the UK, Lloyd et al (1996) compared 62 genetic counsellees (with a family history of breast cancer) with a matched group of attenders at a general practitioner's (GP) surgery. They found these two groups of women to be similar in terms of the outcome measures used and concluded that the risk of breast cancer was not predictive of psychological morbidity. In this study, risk perceptions were recorded before counselling, but 58% of the women self-reported that they had previously overestimated their risk. Assessed after genetic counselling, they were found more likely to underestimate (48%) than overestimate their risk (18%).

*Received 15 January 1998*
*Revised 4 June 1998*
*Accepted 8 July 1998*

*Correspondence to:* A Cull

This paper presents an interim analysis of a subset of data from the first cohort of women attending the clinic to address three main questions:

(1) What were the characteristics of the women who presented at the clinic? In particular, did they overestimate their risk and were they highly anxious women?
(2) Did counselling influence their perception of their risk of developing breast cancer?
(3) Did cancer risk counselling cause distress?

The study afforded the opportunity for exploring some potentially explanatory intervening variables to account for individual differences in risk perception and distress, i.e. beliefs about control of health and coping style in relation to information about threat to health. Preliminary findings are reported. Interpretation of the data presented require some understanding of the clinical context from which they were collected.

### Clinic setting

A multidisciplinary clinic was set up in Edinburgh in the autumn of 1992 to provide genetic counselling and breast screening for young (age <50) women with a family history of breast cancer. The clinic is run in a breast screening centre in the community and at the time of the study was staffed by consultants in genetics and breast surgery, a senior registrar in oncology with training in genetics, a surgical registrar conducting research in genetics for an MD thesis and a specialist nurse. The clinic was funded from a variety of sources to provide a research-based service of which this psychological assessment was an integral part.

The criteria for referral at the time of this study were: (1) a first-degree relative with bilateral breast cancer *or* breast and ovarian cancer *or* breast cancer diagnosed at age < 50 years or (2) two first-degree relatives with breast/ovarian cancer at any age or (3) a male first-degree relative with breast cancer. In practice, referrals were accepted of any woman with a history of concern to the referring agent. Of the first 200 women, the majority (> 70%) were referred from hospital clinics. As the clinic became known, the proportion of referrals from GPs increased (to 55% of the total sample).

Women were counselled individually. At the time of this study, two clinic appointments were usually offered. At the first, a detailed family history was taken and discussed with the geneticist, who gave some general educational information about breast cancer genetics. Clinical examination and mammography (where appropriate) were undertaken and women were offered training in breast self-examination. Opportunity was given for breast cancer worries to be discussed with the breast surgeon/oncologist. Family history data were then verified (i.e. through hospital and public records by pedigree workers) and reviewed by clinic staff at a case conference where risk estimates were assigned with reference to epidemiological data (Claus, 1991) and consensus established on the advice to be given. At their second clinic visit women were given this empiric risk estimate and plans for their future management and subsequent follow-up were discussed with them and subsequently communicated to their GPs by letter. Those not at significantly increased risk were discharged.

### PATIENTS AND METHODS

### Sample

A consecutive series of 486 women newly referred to the clinic over a 27-month period were invited to take part in the study.

### Measures

#### *Risk estimate*

Women were asked to select from 12 response categories the ratio (e.g. 1 in 2 to 1 in 100) which they believed to be (a) the risk for a woman in the general population and (b) their own lifetime risk of developing breast cancer (Evans et al, 1993, 1994).

#### *Risk factors*

Women were asked to identify any of a list of nine factors that they believed would increase the risk of breast cancer (adapted from Fallowfield et al, 1990). The factors were: being single, married without children, married with children, not having breastfed, taking the oral contraceptive pill, having relatives with breast cancer, being past the menopause, having been hit in the breast and stress.

#### *Psychological distress*

The Spielberger state–Trait Anxiety Inventory (STAI, Spielberger, 1983) was used to measure anxiety proneness (trait) and current levels of generalized anxiety (state). Knight et al (1983) collected STAI data from a general population sample in an area of New Zealand with a strong history of immigration from Scotland. The STAI trait and state anxiety scores, which are presented by age (in 10-year bands) and sex, offer more appropriate reference data for this study than those in the STAI manual, which are derived from employees in the US Federal Aviation Administration. Reference data are also available from a large series of women attending for breast screening and for whom STAI trait and state anxiety scores are presented by age (in 10-year age bands from 30–69) and separately for women with normal breasts vs benign disease (Morris and Greer, 1982). More recent trait anxiety data are available from women aged > 50 in the tamoxifen prevention trial and from women with and without a family history of breast cancer undergoing routine screening (Thirla et al, 1996).

The General Health Questionnaire (GHQ)-30 was used to screen for clinically significant levels of psychological distress and dysfunction (Goldberg and Williams, 1988). The manual for this instrument provides extensive comparative data derived from population surveys.

#### *Psychological factors*

The Health-related Locus of Control Scale (Wallston et al, 1978) was used to assess the extent to which the women attributed their health to internal (i.e. own behaviour), external (e.g. doctors) or chance factors. The nine items with the highest item – subscale correlations were selected (Marks et al, 1986). Although no descriptive data were available for comparison, this short form allowed the role of locus of control to be explored while keeping the burden on respondents to a minimum. The Miller Behavioural Style Scale (Miller, 1987) was designed to assess the propensity of people to seek out (‘monitor’) or avoid (‘blunt’) information about threatening events. The short form presents two scenarios from which respondents select their most likely reaction from a fixed choice of ‘monitoring’ and ‘blunting’ responses. Limited comparative data using this version are available from small samples of students and patients with recurrent cancer (Steptoe, 1989). The short form was again considered adequate for exploratory analysis of the role of these constructs within this study.

Data about the women’s health care attitudes and behaviour, which were also collected, will be reported elsewhere.

*© Cancer Research Campaign 1999*

### Procedure

The assessment package was posted to women with their clinic appointment and returned when they attended the clinic. Exceptions were the baseline state anxiety and GHQ-30, which were administered at clinic before the consultation. Risk estimate, state anxiety and GHQ-30 were reassessed at clinic after the second consultation at which risk counselling was undertaken. The measures were again sent to women for completion prior to their annual follow-up at the clinic.

### Statistical analysis

Descriptive statistics were generated to describe the study population. Anxiety and distress data from the same patients on two occasions were compared using paired *t*-tests. Comparisons between two independent samples were made using two-sample *t*-tests. Personal risk estimates (transformed from ratio to percentage risk) from the same patients on two occasions were compared by the Wilcoxon matched-pairs test. Risk estimates from independent groups were compared by the Kruskal–Wallis test. The associations between explanatory variables and ordered groups based on the accuracy of patients' personal risk estimates (under–, close, overestimates) were examined using the non-parametric trend test (Cuzick, 1985). The chi-squared test for trend was used to compare proportions across these ordered groups. Spearman's rank correlation coefficient ($r_s$) was used to measure the association between change in risk estimate and change in GHQ score. The gamma statistic *G* was used as a measure of association for ordered variables when these data were cast in the form of a contingency table. Multiple logistic regression, with backward stepwise selection, was used to examine which variables were predictive of distress, using $P \leq 0.05$ as the criterion for keeping variables in the model. Previous models in the stepwise procedure were examined to assess the justification for retaining exploratory variables, i.e. locus of control, monitoring/blunting in future studies. The data were analysed using the Stata statistical package (Stata Corp., 1995)

### RESULTS

### The sample

Four hundred and eighty-six women referred to the clinic between October 1992 and January 1995 were sent baseline assessments, and 481 (99%) returned them when they attended the clinic. There are variable numbers of missing data per item in the forms returned over the three assessment points. For clarity, the denominator is therefore specified throughout. Baseline data were analysed to assess whether the characteristics of those attending the clinic changed year by year. In the absence of statistically significant time trends, the sample was treated as a single cohort.

At the time of data analysis, a number of women were no longer in the study population. Of the original sample attending the clinic, 136 women (28%) were discharged. Ninety-five of them were not at sufficiently increased risk to warrant surveillance and, of these ten were discharged after the first clinic visit and 85 after the second. Twenty-eight women were discharged because of their age, a further 12 were discharged to the IBIS (International Breast Cancer Intervention Study) trial and one woman was discharged following medical investigation. Of the original sample, 69 (14%) failed to attend subsequent clinics: five of them withdrew from the clinic altogether, 30 failed to attend for follow-up visits in the time frame of this study and 34 were lost to follow-up, for example moved away or administrative failure. Consequently, at the time of data analysis 281 (58% of the original sample) were being kept under surveillance but they were at different stages of follow-up. The numbers of women for whom data were available at the time of analysis were therefore variable across the assessment points. The sample size is specified throughout.

The mean age of the women presenting to the clinic in the study period was 39.6 years ($n = 481$, s.d. = 9.2).

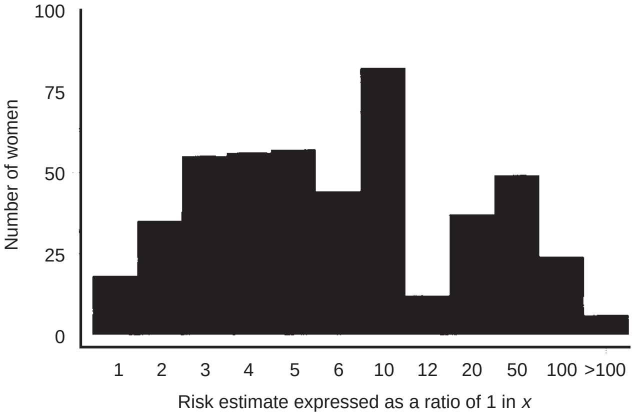

**Figure 1** Women's baseline estimates ($n = 475$) of their own lifetime risk of developing breast cancer

### Preclinic risk estimate

#### *Personal risk estimate*

At baseline, data were available from 475 women to indicate their perception of their risk of developing breast cancer. Eighteen women (4%) believed it inevitable they would develop breast cancer. Thirty women (6%) set their risk as ≤ 1:100. The distribution of risk estimates between these extremes is shown in Figure 1. Sixty-three percent set their own risk at ≥ 2 × general population risk whatever they believed that to be. Thirty-one per cent believed their risk to be equal to or greater than the general population risk by a factor of < 2. Surprisingly, 33 women (7%) set their own risk lower than the general population.

#### *Counselled risk*

The risk ratio assigned by staff at the clinic ('counselled risk') was available from the case notes of 458 women at the time of this analysis. Four women (1%) were given a risk estimate of 1 in 2 and 77 women (17%) were given a risk estimate of less than 1 in 10, i.e. similar to the risk of the general population. Between these extremes, the risk ratios given to those attending the clinic were: ≥ 1 in 3, $n = 92$ (20%); ≥ 1 in 4, $n = 122$ (27%); ≥ 1 in 5, $n = 87$ (19%); ≥ 1 in 10, $n = 76$ (17%).

#### *Accuracy of personal risk estimate before counselling*

The counselled risk and baseline personal risk estimate were both available for comparison for 450 women. Relative to the counselled risk, 65 women (14%) had initially grossly overestimated their risk, i.e. by a factor of ≥ 2 ('overestimators'), and 174 women (39%) had underestimated to the same degree, i.e. ≤ 0.5 ('underestimators'). Most women 47% ($n = 211$) fell between these limits

© Cancer Research Campaign 1999

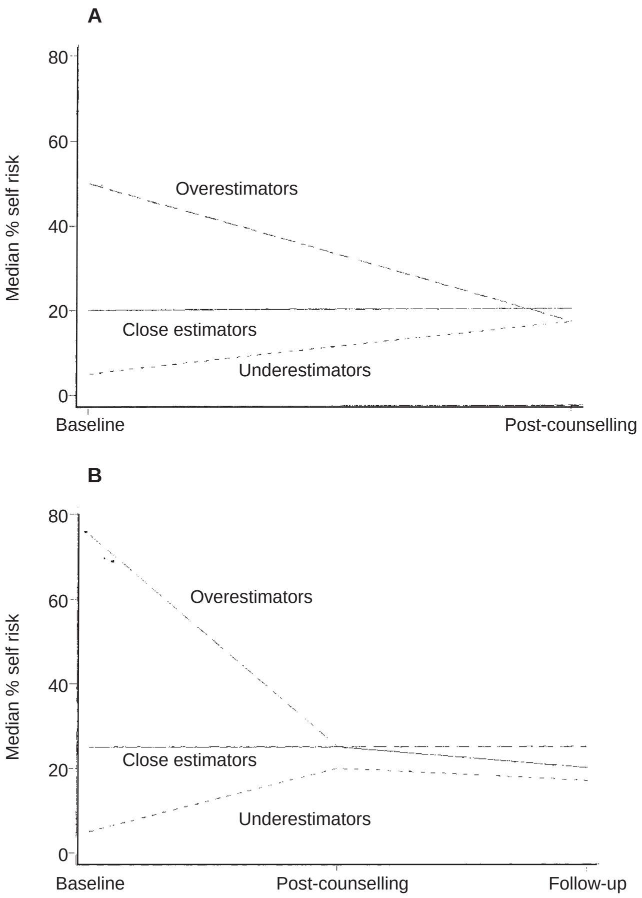

**Figure 2** (**A**) Risk estimates before and after counselling for over-, close- and underestimators (*n* = 363). (**B**) Risk estimates at baseline, post-counselling and annual follow-up for over-, close and underestimators (*n* = 171)

**Table 1** Age and psychological characteristics of the sample as a whole and by accuracy of initial self risk estimate

| | Under | Close | Over | Whole sample |
|---|---|---|---|---|
| *n* age | 174 | 207 | 65 | 481 |
| Mean (s.d.) | 38.1 (8.4) | 39.1 (8.7) | 43.0 (10.5) | 39.6 (9.2) |
| Trait anxiety (*n*) | 174 | 208 | 64 | 475 |
| Mean (s.d.) | 38.8 (9.0) | 39.2 (8.7) | 42.9 (9.9) | 39.6 (9.1) |
| State anxiety (*n*) | 168 | 205 | 65 | 472 |
| Mean (s.d.) | 34.8 (9.4) | 35.3 (9.3) | 36.8 (10.4) | 35.4 (9.5) |
| Locus of control[a] | | | | |
| Internal (*n*)[b] | 174 | 209 | 65 | 478 |
| Mean (s.d.) | 13.7 (2.8) | 13.3 (2.6) | 13.4 (3.1) | 13.4 (2.7) |
| External (*n*)[c] | 174 | 208 | 64 | 475 |
| Mean (s.d.) | 6.8 (3.1) | 6.9 (3.2) | 7.8 (3.5) | 7.0 (3.3) |
| Chance (*n*)[d] | 173 | 208 | 65 | 475 |
| Mean (s.d.) | 7.7 (3.2) | 8.5 (2.9) | 8.2 (3.2) | 8.2 (3.0) |
| Monitoring (*n*)[e,f] | 173 | 211 | 65 | 479 |
| Mean (s.d.) | 3.6 (1.7) | 3.8 (1.6) | 3.9 (1.7) | 3.8 (1.7) |
| Blunting (*n*)[e,g] | 173 | 211 | 65 | 479 |
| Mean (s.d.) | 2.0 (1.2) | 1.9 (1.2) | 2.0 (1.4) | 1.9 (1.2) |

[a]Wallston and Wallston (1978) NB: based on six items per subscale vs three items per subscale for scores in Table 1. [b]Internal, mean = 25.1 (4.9). [c]External, mean = 20.0 (5.2). [d]Chance, mean = 15.6 (5.8). [e]Steptoe (1989). [f]Monitoring: students, mean = 4.7 (1.8); cancer patients, mean = 4.5 (2.0). [g]Blunting: students, mean = 1.4 (1.3); cancer patients, mean = 3.0 (1.6).

and for the purposes of this analysis were designated ‘close estimators’. Thirty women (7%) were exactly accurate in their initial risk estimate, i.e. 1 in 12 (*n* = 2), 1 in 5 (*n* = 16), 1 in 4 (*n* = 11) and 1 in 3 (*n* = 1).

### Impact of counselling on personal risk estimate

Immediately after risk counselling at the clinic, data were available from 363 women who could be categorized on the basis of the accuracy of their baseline risk estimates (over-, close and underestimators). Pre- and post-counselling risk estimates were compared for each category (Figure 2A).

Overestimators (*n* = 49) showed significantly lower risk estimates after counselling ($z = 5.69$, $P < 0.0001$), but they still tended to overestimate their risk relative to the counselled risk ($z = -2.60$, $P < 0.01$). Twelve women persistently overestimated their risk (≥ 2 × counselled risk) before and after counselling.

As a group, underestimators (*n* = 132) reported significantly higher risk estimates after counselling ($z = -8.01$, $P < 0.0001$), but they continued to underestimate relative to the counselled risk ($z = 7.37$, $P < 0.0001$). Sixty women underestimated their risk (≤ 0.5 × counselled risk) at both assessment points.

Women with close estimates at baseline (*n* = 182) tended to report lower risk estimates after counselling ($z = 1.74$, $P = 0.08$). After counselling, their risk estimates were on average significantly lower than the counselled risks they had been given ($z = 2.60$, $P < 0.01$). After counselling, 11 women overestimated (≥ 2×) and 35 underestimated (≤ 0.5×) their risk relative to the counselled risk.

Data were available from 171 women at all three time points, i.e. baseline, post-counselling and follow-up. This subset of data, categorized by the accuracy of the women’s initial risk estimates, is shown in Figure 2B. A significant shift in risk estimates, in the direction of increasing accuracy from baseline to post-counselling, was observed for both the overestimators ($z = 3.7$, $P < 0.0002$) and underestimators ($z = -6.4$, $P < 0.0001$). There was no significant difference between post-counselling and follow-up risk estimates for underestimators or close estimators. Overestimators showed a significant shift ($z = -2.8$, $P < 0.03$), indicating that their risk estimates were increasing over time after risk counselling. Two of the 171 women consistently overestimated their risk at each time point by a factor of ≥ 2, and 17 (10%) consistently underestimated their risk to the same degree over the three assessments.

Some additional analyses were undertaken to assess the representativeness of these findings. Three subsets of data were compared from those who had (1) baseline data only, (2) baseline and post-counselling data only and (3) data available at all three time points. These subsets differed in the median risk estimate at baseline ($\chi^2 = 8.11$, d.f. = 2, $P = 0.02$). Overestimators in subset 3 had much higher risk estimates than those in the other two subgroups ($\chi^2 = 13.03$, d.f. = 2, $P = 0.002$), which inflated the median risk in that subgroup. Precounselling, 9 of the 18 overestimators in subset 3 had believed it inevitable that they would develop breast cancer.

 *© Cancer Research Campaign 1999*

**Table 2** Spielberger Trait and State Anxiety Scores for the study sample with comparative published data

| | *n* | Mean | s.d. | *t* | d.f. | *P* |
|---|---|---|---|---|---|---|
| Trait anxiety | | | | | | |
| Study sample | 475 | 39.6 | 9.1 | | | |
| General population[a] | 586 | 36.9 | 8.9 | 4.9[c] | 1059 | <0.001 |
| Breast screening[b] | | | | | | |
| Benign disease | 350 | 40.2 | 9.3 | –0.9[c] | 823 | N.S. |
| Normal | 282 | 40.4 | 8.7 | –1.2[c] | 755 | N.S. |
| State anxiety | | | | | | |
| Study sample | | | | | | |
| Baseline | 472 | 35.4 | 9.5 | | | |
| Post-counselling | 384 | 33.7 | 9.8 | 3.1[d] | 383 | <0.003 |
| General population[a] | 579 | 33.5 | 8.6 | 3.4[e] | 1049 | <0.001 |
| Breast screening[b] | | | | | | |
| Benign disease | 317 | 41.5 | 11.9 | –8.0[e] | 787 | <0.001 |
| Normal | 274 | 39.1 | 10.8 | –4.9[e] | 744 | <0.001 |

[a]Knight et al, 1983. [b]Morris and Greer, 1982. [c]Two-sample *t*-test from comparison with mean trait anxiety score. [d]Paired *t*-test of baseline and post-counselling state anxiety scores. [e]Two-sample *t*-test from comparison with mean state anxiety score at baseline.

**Table 3** Change in risk estimate and change in GHQ-30 scores, post-counselling from baseline ($n$ = 368)

| Change in risk estimate (post-counselling from baseline | Change in GHQ score (post-counselling) from baseline: Decrease ≥3 | No change | Increase ≥3 | *n* |
|---|---|---|---|---|
| Decrease | 32% | 56% | 12% | 142 |
| No change | 26% | 52% | 21% | 80 |
| Increase | 29% | 53% | 17% | 146 |
| Whole sample | 30% | 54% | 16% | 368 |

Gamma statistic; $G$ = 0.07; $P$ <0.95.

**Table 4** Logistic regression coefficients (and s.e.s) from analysis of the psychological distress score (GHQ-30 score > 5) after risk counselling ($n$ = 363 women)

| | Regression coefficient | s.e. | *z* | *P* | 95% CI |
|---|---|---|---|---|---|
| Age | –0.044 | 0.015 | –2.85 | <0.004 | –0.074 to –0.014 |
| Baseline GHQ | 0.082 | 0.020 | 4.03 | <0.001 | 0.042 to 0.122 |
| Constant | –0.001 | 0.597 | | | |

### Factors influencing accuracy of initial risk perception

At baseline ($n$ = 480), the median number of correct responses to the nine items concerning putative risk factors for breast cancer was 5. Ninety-seven per cent of the women recognized that having relatives with breast cancer was a risk factor. The next most frequently cited risk factor was stress (48%). Overestimators were more likely to identify stress (62%) as a risk factor (cf. 46% of close estimators and 47% of underestimators).

Age and scores for trait and state anxiety, locus of control and coping style (monitoring/blunting) collected at baseline are summarized for the sample as a whole and separately for over-, close and underestimators (Table 1).

The accuracy of women's initial risk estimates appeared to be related to age ($z$ = 2.97, $P$ < 0.005) and trait anxiety ($z$ = 2.52, $P$ < 0.01) but unrelated to state anxiety ($z$ = 1.47, $P$ = 0.14). Older women and those with higher trait anxiety were more likely to overestimate their risk. Those who overestimated their risk tended to have higher scores on the external locus of control scale, i.e. they were more likely to believe that their health depended on others, but this did not reach statistical significance.

### Anxiety

### *Precounselling*

The sample as a whole exhibited a higher mean trait anxiety score (Table 2) than that derived from women in a general population sample (Knight et al, 1983) but not significantly different from scores reported from two samples of women attending a breast screening clinic (Morris and Greer, 1982). Baseline state anxiety scores collected when women first attended our clinic were significantly higher than the data from the general population but significantly lower than the data from the screening clinic sample (Table 2).

### *After counselling*

State anxiety scores were available both at baseline and post-counselling for 384 women. After counselling, state anxiety scores were significantly *lower* than baseline scores for the same women (Table 2). These post-counselling scores were lower than scores from Morris and Greer's (1982) screening clinic sample (benign disease: $t$ = – 9.5, d.f. = 699, $P$ < 0.001; healthy women: $t$ = –6.7, d.f. = 656, $P$ < 0.001) and similar to Knight et al's (1983) general population data (Table 2).

Examined separately, under-, close and overestimators all showed on average a reduction in state anxiety scores after counselling but the difference was statistically significant only for overestimators ($t$ = –2.38, d.f. = 49, $P$ < 0.03). No significant relationship was observed between change in risk estimate and change in state anxiety score from baseline to post-counselling ($r_s$ = 0.05, $P$ = 0.31). Among women assessed at annual follow-up, state anxiety scores tended to be on average lower than at baseline ($t$ = –1.70, d.f. = 179, $P$ = 0.09). For under-, close and overestimators examined separately there were no significant differences between state anxiety scores at baseline and at annual follow-up.

### Psychological distress (GHQ-30)

### *Precounselling*

Four hundred and eighty-one women completed the GHQ-30 at baseline. Their mean score was 4.5 (s.d. = 6.2). Data were compared with general population data derived from respondents to a health and lifestyle survey in the UK (Cox et al, 1987). The mean GHQ score for 777 women in the survey aged 35–44 years was 3.8 (s.e. = 0.19). Younger (age 25–34 years) and older (age 45–54 years) women in this survey had GHQ scores that were similar to our sample: mean score = 4.4 (s.e. = 0.21) and 4.6 (s.e. = 0.21) respectively.

One hundred and forty-four of our sample (30%) scored above the cut-off (5/6) recommended for identifying case-level distress. The percentages of women in the general population survey scoring >5 were: 25–34 years = 34%; 35–44 years = 28%; 45–54 years = 35%.

© Cancer Research Campaign 1999

GHQ data analysed separately for 174 under-, 211 close and 65 overestimators showed no significant differences among the three groups at baseline ($z$ = 1.45, $P$ < 0.15), and the proportion of women exhibiting case-level distress was similar, i.e. 30% vs 28% vs 34% ($\chi^2$ for trend = 0.11, d.f. = 1, $P$ < 0.80).

### *After counselling*

In all, 385 women completed the GHQ-30 before and after risk counselling. Their mean score after counselling (mean = 3.1, s.d. = 4.9) was significantly lower (paired $t$ = 3.6, d.f. = 384, $P$ = 0.0004) than the mean score returned by the same women at baseline (mean = 4.3, s.d. = 6.1). From this group, there were 169 women for whom data were available at annual follow-up. They showed no significant differences between mean GHQ scores at baseline (mean = 4.2, s.d. = 6.0) and annual follow-up data (mean = 4.1, s.d. = 6.2; paired $t$ = 0.14, d.f. = 168, $P$ = 0.90).

### Changing risk estimates and distress

A key concern was that, if counselling increased women's perception of their risk, this would cause distress. Full data were available for 368 women assessed before and after risk counselling. There was no evidence of an association between change in risk estimate and change in GHQ scores ($r_s$ = 0.04, $P$ < 0.50). The sample was divided on the basis of whether the woman's risk estimate increased, remained the same or decreased after counselling relative to her baseline estimate. Changes in GHQ-30 scores of ≥3 points in either direction were recorded and tabulated against change in risk estimate of ≥1% point in either direction (Table 3).

Again there was no evidence that increasing estimate of risk was associated with increased distress. Thirty per cent of this sample were less distressed after risk counselling, but this was not directly related to having a lower risk estimate.

### Predicting post-counselling distress

Clinically it is important to be able to predict which women are likely to show case-level distress so that appropriate intervention can be offered. A backward stepwise logistic regression analysis was undertaken using data from 363 women to determine the contribution of the variables assessed at baseline to predicting a GHQ score of >5 after risk counselling. Variables entered into the model were: age, number of risk factors correctly identified, Spielberger anxiety scores, baseline GHQ-30 and scores from the locus of control and monitoring/blunting scales. Accuracy of initial risk estimate and change in baseline risk estimates were examined in earlier models which included the above variables, but neither was found to be associated with an increased risk of showing case-level distress. The final model is shown in Table 4.

Younger age and higher GHQ score at baseline were associated with an increasing risk of case-level distress, as assessed by the GHQ, after risk counselling.

At this stage in our understanding we wanted to clarify whether any other variables suggested an effect warranting further study in the future. In this analysis, we therefore examined the order in which variables were removed from the model. The model before that reported in Table 4 included one locus of control variable, i.e. external locus of control. The coefficients for age and GHQ in this model are similar in magnitude to those in the final model reported. Increasing external locus of control tended to be associated with an increasing risk of exhibiting case-level distress, although this was not statistically significant at conventional levels ($P$ < 0.11).

### DISCUSSION

Before this familial breast cancer clinic was set up, women with a family history of breast cancer of concern to them or their GP had been referred to a symptomatic breast clinic. This offered clinical examination and mammographic screening but risk assessment was not discussed. These women were referred on when this clinic was set up. The proportion of women referred direct from GPs increased as the clinic became established, but there was no evidence that this shifting practice made a significant difference in terms of time trends in the variables of interest for this study. Compliance with the baseline assessment was excellent so the data presented adequately describe the first cohort of women to attend this clinic.

### Do breast cancer genetic counselling clinics attract women who grossly overestimate their risk and who are highly anxious?

The first cohort of women who attended this clinic in SE Scotland were aware of their increased risk relative to the general population (two-thirds of them set themselves at twice the population risk, whatever they conceived that to be) but they were more likely to underestimate their risk (39%) than to overestimate it (14%). These data echo the experience of the clinic in Manchester (Evans et al, 1993).

It is important to avoid ascribing a spurious accuracy to the women's risk estimates collected using this methodology, the limitations of which we fully acknowledge. We also recognize that variations across studies in the way in which risk is calculated and the accuracy of women's estimates defined make comparison difficult. Nonetheless, there is a stark contrast between our data and Lerman et al's trial (1995) of breast cancer risk counselling, in which two-thirds of the women grossly overestimated their own cancer risk. There may be important cross-cultural differences in cancer risk perception but it is also important to be aware of how samples are derived. In Lerman's trial, women had been identified through a relative currently receiving treatment for breast cancer. It is likely that this would have increased their sense of their own susceptibility.

There is a lack of consensus among clinicians about how best to communicate information about risk, e.g. in words (e.g. high, low) vs numbers (e.g. ratios or percentages). We elected to use ratios as the method of assessing risk estimate because the general population risk is commonly expressed in those terms (although personal risk information is typically given in a variety of ways even within a single consultation) and, because of this, was the form of the only comparable data available at that time (Evans et al, 1993). Epidemiological data continue to be analysed to provide genetic counsellors with more accurate numerical values for relative (Pharoah et al, 1997) and absolute risk (Pharoah and Mackay, 1998). It remains unclear what these various numerical values of risk estimates mean to the women to whom they are assigned (Hallowell and Richards, 1997). Psychosocial research suggests that lay understanding of genetic risk derives more from concepts of family relationships than from scientific genetics (Richards and Ponder, 1996). More detailed reviews of the issues in cancer genetic risk counselling have recently been published (Bottorff et

© Cancer Research Campaign 1999

al, 1998; Cull, 1998). Further research is needed to clarify how different degrees of risk are communicated and can best be understood in this setting.

In terms of knowledge of risk factors, the women in this sample were better informed than Fallowfield et al's (1990) sample of women attending for routine screening, but some misconceptions prevailed – for example, half of our sample believed that stress has been shown to increase the risk of breast cancer. It was beyond the remit of this study to assess how that belief impinged on their view of their own susceptibility but this may be important in influencing how they elect to manage their own breast cancer risk.

This sample did have somewhat higher trait anxiety scores than the mean for a general population sample (Knight et al, 1983) but no higher than for women who elected to attend for routine mammography (Morris and Greer, 1982). More recently, Thirlaway et al (1996) reported trait anxiety scores from participants in the Tamoxifen Prevention trial and women in the National Breast Screening Programme (NBSP), separating those who did/did not have a family history of breast cancer and those who were/were not aware of the population risk of the disease. The 24 women in the NBSP with a family history who were aware of the population risk were the most anxious (mean trait anxiety score = 45.5, s.d. = 9.5). The other groups of women had anxiety scores similar to our data. State anxiety scores in our sample were no higher than for women attending for routine mammography and were unrelated to the women's baseline risk estimates.

There was then no evidence that this genetic risk counselling clinic was attended by women who grossly overestimated their risk. On the contrary, they were almost three times more likely to underestimate than overestimate their risk. Nor was there evidence that they were more anxiety prone or acutely anxious than other women who attended for routine breast screening, although those who did overestimate their risk were more anxiety prone. The characteristics of those attending such clinics may change in future as public awareness increases and as referral criteria are more strictly applied to optimize access to services for those at highest risk. It is likely to be important for effective counselling to take account in the clinic of women's initial perception of their risk and the factors that influenced them to attend, e.g. a close relative recently diagnosed with breast cancer, as counselling may need to be tailored accordingly.

### Does risk counselling influence risk perception?

From the point of view of service evaluation, an important finding of this study was that the risk counselling offered increased the accuracy of the women's perceptions of their own risks of developing breast cancer. Although risk estimates have limitations as a primary outcome measure, these data are encouraging in the light of the results of the only randomized trial of breast cancer risk counselling reported to date (Lerman et al, 1995). The risk estimates of the two-thirds of the women who grossly overestimated their risk initially were not modified by the counselling offered in either arm of the trial.

We have, however, no room for complacency. The overestimators in our sample reduced their risk perceptions significantly after counselling, but they continued to overestimate their risk to a statistically significant degree relative to the counselled risk and their estimates appeared to be increasing again on longer term follow-up. The underestimators as a group did increase their risk estimates significantly after counselling, but they continued to underestimate relative to the counselled risk. Applying these findings in the clinic it would seem to be important to identify and understand the basis of such persistent misperceptions in order to find an effective means of correcting misunderstandings.

Care is clearly needed in the analysis and interpretation of a dataset with so many missing data. Some women were discharged because they were not at sufficiently increased risk to warrant surveillance, some were lost to follow-up and others had not been followed up by the time this analysis was undertaken. We were unable to demonstrate any significant time trends in the presenting characteristics of women attending the clinic in successive years. Comparing the data from women responding at all three assessment points (Figure 2B) with the subsets of data for women assessed pre- and post-counselling or at baseline only, this subset of women displayed a higher median risk estimate at baseline, most notably among the overestimators. The numbers are small, i.e. there were 18 overestimators for whom annual follow-up data were available. Half of them had reported at baseline that they believed that it was inevitable that they would develop breast cancer (risk estimate = 100%), thereby inflating the median risk in that subset of data. It is particularly reassuring then that, even in this subset of women with more extreme views, the counselling available was effective in reducing risk estimates in a way that was sustained to annual follow-up.

This cohort had the benefit of a model of service delivery that was unsustainable in the face of growing demand for service. They had a first clinic visit which gave an opportunity for their family history and cancer related concerns to be discussed and for general information to be given about breast cancer genetics. There was time for the women to assimilate this before the second consultation about their personal risk and risk management. We are continuing to assess the impact of evolving models of service delivery in SE Scotland on women's estimates of their risk of developing breast cancer and to develop methods of assessing their understanding of the concepts involved, which may be more clinically informative than risk estimates alone (Cull et al, 1998).

### Does cancer risk counselling cause distress?

Mean state anxiety and GHQ scores were significantly lower after counselling than before, confirming what many women volunteered at clinic, i.e. that they felt reassured by being able to attend. There was no evidence that counselling caused anxiety or distress to those who were made aware that their risk of developing breast cancer was greater than they had previously thought.

The risk of being significantly distressed after risk counselling was higher among women with higher GHQ scores at baseline. This is a reminder that among those who attend a clinic like this will be a proportion of women who are already distressed by their experience of cancer in their families and whose concerns may warrant particular attention. The role of beliefs about control of one's health in the face of inherited susceptibility to a life-threatening illness warrants further study. Our preliminary findings suggest that believing that external or chance factors (rather than one's own actions) control health may be associated with a sense of greater risk and with greater distress. Elucidation of the relationship between these factors may suggest effective methods of intervention to reduce distress. For example, behaviours that increase the individual's sense of control over their health may be a helpful coping strategy in this context (Ingledew et al, 1996).

In conclusion, it was reassuring in the light of the US experience to find that the counselling offered in this clinic was effective in

*© Cancer Research Campaign 1999*

increasing the accuracy of risk perceptions without causing distress to those who initially underestimated their risk. It is worrying that so many inaccuracies persisted, particularly as the demand for service has required a reduction in consultation time in this clinic. There is a need to develop more sophisticated methods of assessing people's understanding of the concepts involved in assigning familial cancer risk. Inaccurate risk perceptions may be attributable to misunderstandings of such complex and probabilistic information but may also reflect contrary beliefs arising from people's personal experience of cancer in the family. Evaluation of alternative models of delivering information about cancer risk should in future include objective assessment of the recipients' understanding of key items of the information given. Future studies also need to consider the influence of personal experience of cancer in the family on preconceptions about personal risk and levels of distress among those attending familial breast cancer clinics.

### REFERENCES

Bottorff JL, Ratner PA, Johnson JL et al (1998) Communicating cancer risk information: the challenges of uncertainty. *Patient Education Counselling* **33**: 67–81

Claus EB, Risch NK and Thomson WD (1991) Genetic analysis of breast cancer in the cancer and steroid hormone study. *Am J Hum Genet* **48**: 232–242

Cox B, Blaxter M, Buckle A, Fenner NP, Golding J, Gore M, Huppert F, Nickson J, Roth M, Stark J, Wadsworth M and Wichelow M (1987) *The Health and Lifestyle Survey*. Cambridge: Health Promotion Research cited in Goldberg and William (op cit).

Cull A (1998) Psychosocial implications of Progress in Breast Cancer Genetics: uncertainties and Challenges. *Clin Psychol Psychother* **5**: 109–123

Cull A, Miller H, Porterfield T, Mackay J, Anderson EDC, Steel CM and Elton RA (1998) The use of videotaped information in cancer genetic counselling: a randomised evaluation study. *Br J Cancer* **77**: 830–837

Cuzick J (1985) A Wilcoxon-type test for trend. *Stat Med* **4**: 87–90

Evans DGR, Burnell LD, Hopwood P and Howell A (1993) Perception of risk in women with a family history of breast cancer. *Br J Cancer* **67**: 612–614

Evans DGR, Blair V, Greenhalgh R, Hopwood P and Howell A (1994) The impact of genetic counselling on risk perception in women with a family history of breast cancer. *Br J Cancer* **70**: 934–938

Fallowfield LJ, Rodway A and Baum M (1990) What are the psychological factors influencing attendance, non attendance and re-attendance at a breast screening centre? *J Roy Soc Med* **83**: 547–551

Gagnon P, Massie MJ, Kash K, Gronert M, Heerdt AS, Brown K, Sullivan MD and Borgen P (1996) Perception of breast cancer risk and psychological distress in women attending a surveillance program. *PsychoOncology* **5**: 259–269

Goldberg D and Williams P (1988) *GHQ: A Users Guide to the General Health Questionnaire*. NFER-Nelson: Windsor

Hallowell N and Richards MPM (1997) Understanding Life's Lottery: an Evaluation of Studies of Genetic Risk Awareness. *J Health Psychol* **2**: 31–43

Ingledew DK, Hardy L, Cooper CL and Jemal H (1996) Health behaviours reported as coping strategies: a factor analytical study. *Br J Health Psychol* **13**: 263–281

Kash KM, Holland JC, Halper MS et al (1992) Psychological distress and surveillance behaviours of women with a family history of breast cancer. *J Natl Cancer Inst* **84**: 24–30

Knight RG, Waal Manning HJ and Spears GF (1983) Some norms and reliability data for the State Trait Anxiety Inventory and the Zung Self Rating Depression Scale. *Br J Clin Psychol* **22**: 245–249

Lerman C, Daly M, Sands C et al (1993) Mammography adherence and psychological distress among women at risk for breast cancer. *J Natl Cancer Inst* **85**: 1074–1080

Lerman C, Kash K and Stefanek M (1994*a*) Young women at increased risk for breast cancer: perceived risk, psychological wellbeing and surveillance behaviour. *Monogr Natl Cancer Inst* **16**: 171–176

Lerman C, Daly M, Masny A et al (1994*b*) Attitudes about genetic testing for breast–ovarian cancer susceptibility. *J Clin Oncol* **12**: 843–850

Lerman C, Lustbader E, Rimer B, Daly M, Miller S, Sands C and Balshem A (1995) Effects of individualised breast cancer risk counselling: a Randomised Trial. *J Natl Cancer Inst* **87**: 286–292

Lloyd S, Watson M, Waites B, Meyer L, Eeles R, Ebbs S and Tylee A (1996) Familial breast cancer: a controlled study of risk perception, psychological morbidity and health beliefs in women attending for genetic counselling. *Br J Cancer* **74**: 482–487

Marks G, Richardson JL, Graham JW and Levine A (1986) Role of health locus of control beliefs and expectations of treatment in adjustment to cancer. *J Pers Soc Psychol* **51**: 443–450

Miller SM (1987) Monitoring and Blunting: Validation of a Questionnaire to Assess Styles of Information Seeking Under Threat. *J Pers Soc Psychol* **52**: 345–353

Morris T and Greer S (1982) Psychological characteristics of women electing to attend a breast screening clinic. *Clin Oncol* **8**: 113–119

Pharoah PDP and Mackay J (1998) Absolute risk of breast cancer in women at increased risk: a more useful clinical measure than relative risk? The Breast (Submitted) Information presented to a Concensus meeting on the management of women with a family history of breast cancer, January 1998, Wellcome Trust: London

Pharoah PDP, Day NE, Duffy S, Easton DF and Ponder BAJ (1997) Family history and the risk of breast cancer: a systematic review and meta-analysis. *Int J Cancer* **71**: 800–809

Richards M and Ponder M (1996) Lay understanding of genetics: a test of a hypothesis. *J Med Genet* **33**: 1032–1036

Spielberger C (1983) *Manual for the State Trait Anxiety Inventory*. Consulting Psychologists Press: Palo Alto, CA

StataCorp (1995) *Stata Statistical Software: Release 4.0.* Stata Corporation: College Station, TX

Steptoe A (1989) An abbreviated version of the Miller Behavioural Style Scale. *Br J Clin Psychol* **28**: 183–184

Thirlaway K, Fallowfield L, Nunnerley H and Powles T (1996) Anxiety in women 'at risk' of developing breast cancer. *Br J Cancer* **73**: 1422–1424

Wallston KA and Wallston BS (1978) Development of the Multidimensional Health Locus of Control Scales. *Health Ed Mono* **6**:(2) 160–170

© Cancer Research Campaign 1999
